# Media Reporting of Health Interventions: Signs of Improvement, but Major Problems Persist

**DOI:** 10.1371/journal.pone.0004831

**Published:** 2009-03-18

**Authors:** Amanda Wilson, Billie Bonevski, Alison Jones, David Henry

**Affiliations:** 1 Discipline of Clinical Pharmacology, School of Medicine and Public Health, The University of Newcastle, Newcastle, Australia; 2 Centre for Health Education and Psycho-oncology (CHeRP), School of Medicine and Public Health, The University of Newcastle, Newcastle, Australia; 3 Institute for Clinical Evaluative Sciences, Sunnybrook Health Sciences Centre, Toronto, Canada; Copenhagen University Hospital, Denmark

## Abstract

**Background:**

Studies have persistently shown deficiencies in medical reporting by the mainstream media. We have been monitoring the accuracy and comprehensiveness of medical news reporting in Australia since mid 2004. This analysis of more than 1200 stories in the Australian media compares different types of media outlets and examines reporting trends over time.

**Methods and Findings:**

Between March 2004 and June 2008 1230 news stories were rated on a national medical news monitoring web site, Media Doctor Australia. These covered a variety of health interventions ranging from drugs, diagnostic tests and surgery to dietary and complementary therapies. Each story was independently assessed by two reviewers using ten criteria. Scores were expressed as percentages of total assessable items deemed satisfactory according to a coding guide. Analysis of variance was used to compare mean scores and Fishers exact test to compare proportions. Trends over time were analysed using un-weighted linear regression analysis. Broadsheet newspapers had the highest average satisfactory scores: 58% (95% CI 56–60%), compared with tabloid newspapers and online news outlets, 48% (95% CI 44–52) and 48% (95% CI 46–50) respectively. The lowest scores were assigned to stories broadcast by human interest/current affairs television programmes (average score 33% (95% CI 28–38)). While there was a non- significant increase in average scores for all outlets, a significant improvement was seen in the online news media: a rise of 5.1% (95%CI 1.32, 8.97; P 0.009). Statistically significant improvements were seen in coverage of the potential harms of interventions, the availability of treatment or diagnostic options, and accurate quantification of benefits.

**Conclusion:**

Although the overall quality of medical reporting in the general media remains poor, this study showed modest improvements in some areas. However, the most striking finding was the continuing very poor coverage of health news by commercial current affairs television programs.

## Introduction

The mainstream media are often the first source from which the public, including health professionals, learn about medical advances [1], [2], [3], [4]. It is crucial when dealing with health issues to avoid creating false hope to those most vulnerable, or generating unwarranted pressure on limited healthcare funding for interventions [5], [6]. There is a general expectation that the media will provide accurate, unbiased and complete information. Journalists endeavour to fulfil these expectations. The ethical obligations of media outlets are reflected in advice from the Australian Press Council, which advocates “a conservative, careful approach to health and medical reports” [7]. However, few attempts have been made to examine whether health news reporting follows these recommendations [8].

There is growing realisation of the potential of the media to influence health behaviours [9]. Public health advocates and researchers see a role for the media in conveying important health messages and awareness campaigns including preventative screening, suicide prevention and smoking cessation [10], [11], [12], [13], [14], [15]. As a result media outlets are inundated with sometimes conflicting health information from companies, researchers, institutions, the government and consumers. Yet, there is little or no specialised training available for Australian journalists who are expected to interpret often impenetrable statistics and health jargon.

Until recently, researchers, medical journals and other independent groups have done little to assist journalists interpret scientific developments for the public. To a degree this situation is changing, with the creation of science media centres in the United Kingdom and Australia (www.aussmc.org/index.php; www.sciencemediacentre.org/index.html). Some medical journals provide media releases to accompany the publication of important studies; but doubts have been expressed regarding the quality of these [16]. Pharmaceutical companies and their public relations consultants have active media strategies but these are designed to promote specific products rather than inform the public about health. As a result of these and other factors, health news stories tend to present incomplete information, which is often skewed towards either extreme of the disease process (underemphasised or exaggerated) or commercial product promotion, while complex research data are often misinterpreted or ignored [8], [17], [18], [19].

The Media Doctor web site (mediadoctor.org.au) was launched in 2004 with the aim of providing an objective analysis of the strengths and weaknesses of the health stories appearing in the Australian mainstream media. A secondary aim was to increase the completeness of health stories and, subsequently, health literacy among journalists and media consumers. Media Doctor Australia was initially described in 2005 when the characteristics of the first 100 news stories reviewed were reported [20]. To date, Media Doctor has reviewed over 1200 stories and similar sites have been launched in Canada (www.mediadoctor.ca) and in the USA (www.healthnewsreview.org) [8], [21].

This paper describes a critical review of 1230 stories reviewed by Media Doctor between 2004 and 2008. Differences between health stories have been analysed according to news outlets, media type (online versus print), and over time. Since the first Media Doctor paper [20], health news stories from popular human-interest, current affair-style television programs have been included in the analyses and the quality of their reports is a particular focus of this paper.

## Methods

Media Doctor reviews health news stories published in the Australian commercial and publicly funded media, including newspapers, online news and transcripts of television and radio broadcasts. Stories are gathered by a researcher who systematically searches news internet sites where articles or transcripts are downloaded. Most of these sites have dedicated health pages. Sites without health pages are searched using stem keywords such as ‘health’, ‘test’, ‘research’, and ‘study’. However, it is possible that some relevant stories are missed using these search strategies. Stories are eligible for review if they cover new health interventions for humans, including drugs, surgical procedures, diagnostic tests, and complementary therapies. The stories are seen as a product of the media outlet and are rated in this capacity. Authorship is not a criterion for assessment and although we collect this information, journalists' names are not publically listed on the website. While all stories rated on Media Doctor come from Australian media outlets, they are not limited to local content and include ‘wire’ stories imported from overseas news outlets. Most stories are derived from research-based interventions but this is not an inclusion criterion. Relevant material such as media releases or journal articles are sent with the story to two reviewers.

Media Doctor reviewers include clinicians and researchers who conduct the reviews in a voluntary capacity. Biographical details of reviewers are available on the website. New reviewers participate in an hour long induction session where all aspects of the website and rating instrument are discussed and demonstration ratings of stories are conducted. All reviewers are provided with ongoing email and telephone support as required. All new reviewers are paired with an experienced reviewer for the first year or so of rating. There have been over 20 reviewers during the four years Media Doctor has been operating and 17 of these remain active. Some review occasionally only, on subjects relating to their expertise. A core group of eight has been rating consistently since the site's inception and these reviewers are responsible for the majority of the reviews. Reviewers rate stories independently of each other using validated rating instruments (for medical interventions and diagnostic tests) [20]. The instruments contain 10 items (see Table 1). These are the same items used by media Doctor Canada and Health News Review in the USA.

**Table 1:** 10 Criteria used to rate news articles about medical interventions.

Rating Criteria*: The extent to which the story:
**1.** **Reported the novelty of the intervention**
**2.** **Reported the availability of the intervention**
**3.** **Described the treatment or diagnostic options that are available**
**4.** **Avoided elements of disease mongering**
**5.** **Reported evidence supporting the intervention**
**6.** **Quantified the benefits of intervention**
**7.** **Described the harms of intervention**
**8.** **Reported on the costs of intervention**
**9.** **Consulted with independent expert sources of information**
**10.** **Went beyond any available media release.**

* Stories are marked ‘satisfactory’, ‘not satisfactory’ or ‘not applicable’. Criteria used to determine scores are available at http://www.mediadoctor.org.au/content/ratinginformation.jsp.

For each news article, the ten criteria are scored as ‘satisfactory’, ‘not satisfactory’ or ‘not applicable’ if a criterion is not relevant. Scores are assigned by each reviewer based on a scoring guide. Total scores (expressed as proportion of items rated ‘satisfactory’) are posted for articles that have seven or more ‘evaluable’ items. Scores are visually depicted on the website using a 1–5 ‘star’ rating along with commentaries from the reviewers. Cumulative scores for the major media outlets are also presented, which provides ongoing feedback on their performance compared with other outlets (http://mediadoctor.org.au/content/media.jsp). Reviewers post their draft reviews in a password-protected area of the website and discrepancies are resolved by consensus. If necessary, a third reviewer is used to settle disagreements. To ensure objectivity, all reviews are screened by a researcher who checks the scores and edits comments. Both reviewers contribute to the comment section, which is used to highlight the strengths of the story, or aspects that could have been improved, including areas not covered in the rating instrument, such as sensationalist language or inappropriate headlines. The turnaround for reviews is approximately two weeks from locating the news story to having it appear on the website.

### Statistical Analysis

Cumulative total satisfactory scores for the nine media outlets were calculated. The media outlets were grouped into four broad categories for the purposes of analysis: Tabloid Newspapers (The Daily Telegraph and Herald Sun), Broadsheet Newspapers (The Australian, Sydney Morning Herald and The Age), Online News Services (ABC Online and ninemsn) and Commercial Current Affairs Television (Today Tonight' Channel 7 and ‘A Current Affair’ Channel 9).

Inspection of the data showed that they were normally distributed, and unweighted cumulative scores were compared between media outlets using analysis of variance (ANOVA). To examine the trend in scores over time we performed linear unweighted regression analyses with time of publication (in days since March 2004) on the horizontal axis and percentage overall satisfactory scores for each article on the vertical axis. Separate regression analyses were performed for online and print media. To compare the proportions that were satisfactory for specific items across the media outlets, Fisher's exact test was conducted. All statistical calculations were made using StatsDirect (version 2.3.6, Stats Direct Ltd, Sale, Cheshire, UK).

## Results

Between March 2004 and July 2008 Media Doctor posted 1230 reviews of health stories from Australian mainstream media. Of these, 613 (50.7%) were about pharmaceutical products, 121 (10%) reported on diagnostic tests, 98 (8.1%) were about surgical procedures, and 387 (31.5) were classified under the heading ‘other’. Stories classified as ‘other’ include complementary and alternative medicines, physiotherapy and dietetics.

### Differences across media outlets

Current trend-lines of scores for individual media outlets are available on the website (http://mediadoctor.org.au/content/media.jsp). The average score for all outlets during the study period was 52% (95% CI 51–53%). Broadsheet newspapers performed best with an average score of 58% (95% CI 56–60%); followed by tabloid newspapers 48% (95% CI 44–52) and online stories 48% (95% CI 46–50); the current affairs television programs scored lowest (average score 33% (95% CI 28–38)) (see Figure S1). The differences in scores across these outlets were statistically significant when assessed by Analysis of Variance (p<0.0001).

We carried out regression analysis of the trends in scores over time for online media outlets (Figure S2). The slope of the regression line was consistent with a small, but statistically significant increase in average score over time. Regression analyses for other forms of media outlets showed no associations.

### Changes in individual item scores over time for all media

An equi-point in data collection of December 2005 was selected to provide two time periods of monitoring with a similar number of articles in each. The average scores for these time periods for each of the 10 rating items are displayed in Table 2. This table illustrates the range and content underpinning the mix of health stories as well as reflecting how well different aspects of the stories are presented. Five items rated under 50% satisfactory: ‘cost’, ‘evidence’, ‘harms’, ‘benefits’ and ‘sources’. Three items (quantification of benefits, the availability of treatment or diagnostic options, and description of harms associated with the intervention) showed significant improvements over the duration of the study (p = 0.007, p = 0.019, p = 0.0005 respectively). Despite the improvement in the way benefits of interventions were reported, it is worth noting that only 36% of stories reviewed presented quantitative data in an adequate manner.

**Table 2:** Mean scores of Rating Instrument items rated satisfactory.

Instrument Items	% Satisfactory	
	2005	2008
Avoided disease mongering	88	89
Novelty of intervention	81	83
Did not rely heavily on media release	73	70
Availability of intervention	53	56
Treatment options available	44	51
Consulted independent expert sources	38	39
Evidence supporting intervention	38	37
Quantified benefits	29	36
Reported costs of intervention	27	36
Described harms of intervention	13	18

### Poor health reporting by commercial human interest programs

As the quantitative data show, television current-affairs programs scored poorly. Some of their stories unashamedly promoted products and a substantial number of them (35%) involved interventions to improve physical appearance: cellulite, wrinkles, body shape and ageing. The fascination for stories about cellulite appears to be confined to current-affairs programs, as no other media outlet covered this topic in our analysis. Our reviewers struggled with gratuitous hyperbole involved in these stories: “After battling cellulite for years…”, “Cellulite may not be life-threatening but…” and “Many women would do anything to get rid of the cellulite”. Unusual and possibly harmful interventions were advocated in these stories. These included: ‘hypoxitherapy’ which involves ‘gentle exercise’ with the offending body parts in a vacuum; ‘lipostabil’ a product not licensed or proven for this sort of cosmetic use; a microwave device ‘biomesosculpture’; and “a new, non-surgical technique called ‘mesotherapy’ in which a cocktail of drugs, vitamins and supplements is injected into the patient”. The cellulite stories scored poorly overall and all were seen as containing strong elements of disease mongering.

More troubling were stories that involved untested cancer treatments or unproven interventions for children with learning or behavioural problems. ‘Today Tonight’ and ‘A Current Affair’ both promoted the Dore Program for learning disorders extolling its virtues with language including ‘cure’, ‘groundbreaking’, ‘transformation’, ‘staggering’ results and a ‘permanent solution’. Despite this, there was no presentation of satisfactory evidence that the program works, nothing about alternative treatments, no information on adverse effects and no attempt to consult an independent expert. The only rating items that scored satisfactory were the ‘availability of treatment’ - which came close to blatant promotion, one story reported on the high cost of treatment. Earlier this year the Dore program went into receivership leaving staff and clients financially disadvantaged; however, neither current affairs program has so far covered this aspect of the story.

Cancer was also the target of stories presenting unconventional treatments. An Australian doctor claimed to cure cancer using ‘ultra high frequency microwave therapy’ along with low dose cyclophosphamide, cystine disulphide or penicillamine (referred to by the practitioner as ‘glucose blocking agents’). While Media Doctor reviewed only two stories on this topic, the current affair programs featured the doctor repeatedly [22]. The campaign in support of this treatment was so intense that the Australian government commissioned an external review, carried out by a specially convened committee of the Australian National Health and Medical Research Council [23]. This found no evidence of efficacy for the procedure. Despite this finding, the Media Doctor website received a large number of responses to our reviews, asking for help in locating this treatment.

Media Doctor has reviewed only a handful of stories from current affairs television programs that presented high quality stories about health. One such example was a story on corrective contact lenses to treat myopia; this rated highly with only one item – ‘evidence’ – scoring not satisfactory. However, the reviewers noted the story discussed planned studies associated with the intervention. The reviewers noted that this was an informative story which “*presented good coverage of the science and alternative treatments*”. It proves these outlets have the potential to cover health issues in a restrained and balanced manner.

## Discussion

After the first 100 Media Doctor reviews, we concluded that the general standards of reporting of medical news in the general press in Australia were poor [20]. Over 1000 articles later, there are some small signs of improvement, but the overall quality remains low. Considered alongside recent reports from Canada and the USA [8], [21], we are forced to conclude that the general media are generally failing to provide the public with complete and accurate information on new medical treatments. However, this analysis shows that the media are capable of improvement. The online news outlets demonstrated an overall improvement of around 5% over the course of the study. There were small improvements in coverage of the following items: the availability of the intervention in Australia; the novelty of the intervention, the cost of the intervention, and the use of independent sources for comment. The areas of significant improvement included the effort made by the journalists to accurately quantify the benefits of the interventions and describe the harms. This is important as it has been pointed out repeatedly that many journalists have difficulty distinguishing between relative and absolute measures of change [24], [25]. The publication of relative risks in general media has resulted in significant numbers of people stopping medications, with potentially harmful impact of that cessation [26], [27], [28], [29].

One media sector that has shown no improvement is the genre of human interest ‘current affairs’ television programs. In Australia these are predominantly aired on commercial channels and their coverage of health news stories largely consists of exaggerated and uncritical endorsement of improbable treatments, including fad diets. It can be argued that when these are directed at relatively harmless conditions, such as cellulite, the stories are unimportant. However, these programs also addressed serious conditions including cancer and behavioral disorders in children. Interventions were portrayed as ‘breakthroughs’ and ‘cures’, no doubt raising false hopes and generating income for the relevant groups of practitioners. This is a source of concern for health, as these programs attract very large audiences with around 2.7 million viewers (17% of the Australian adult population) watching either program each night [30] and have the potential to influence the beliefs and expectations of a substantial portion of the public. There was strong promotional language in many stories and the transcripts on media websites frequently had links to the manufacturers of the ‘treatments’.

There is little in the way of feedback, positive or otherwise, given to journalists and news outlets and none that provides the objective measurements that Media Doctor and similar sites do. Media Doctor has received both negative and positive reactions from journalists. Some have disputed the methodology, such as using the same rating instruments to score television, newspaper and radio stories. We acknowledge limitations and are refining the methodology. We are interviewing a cohort of journalists, editors and producers who are providing feedback on the site and suggesting changes to improve the impact on the media. Journalists and media outlets receive an email alert when their articles are reviewed on the website. Consenting media outlets and journalists are also sent periodic information on their overall ratings compared with other outlets.

The responsibility for accurate health reporting is not solely the province of the media. Researchers and medical journal editors need to provide balanced and accurate media releases on published studies, designed to inform journalists, and through them the public, rather than generate a high media profile for the journal. There is evidence that many journalists feel they lack the medical knowledge to question the authority of experts [2], [31], [32]. Woloshin and Schwartz in their analysis of journal press releases identified problems including the lack of information about study limitations or industry funding. The majority of press releases present data in formats that overemphasize the significance of the findings.[16] There have been repeated calls to limit press releases from early research, such as the kind presented at scientific meetings where the number of presentations that translate into effective treatments are low. [18], [33], [34]

We suggest that a uniformly structured style of media release could be used by medical journals to support journalists and increase quality in reporting of research. The release should address most of the items in the validated Media Doctor rating instrument, such as the novelty of the research, the availability of the intervention including the stage of research and the implications for human application. There should be a clear estimate of when the intervention will be widely available and a rider stating that research at very early stages may never evolve to a treatment phase. The level of evidence presented and study design should be included as well as the number of subjects. Benefits or risks should be quantified in absolute terms. Presenting only relative percentages results in misinterpretation and possible deception. In the interviews described above, Australian health journalists have told of senior management who only deal with relative results, as these provide more sensational stories and many journalists admitted they did not understand the difference between the absolute and relative results. Any adverse events should be noted, as should the potential cost of the intervention especially if this can be compared with existing therapy. All links to industry and all funding should be included. Researchers and independent experts also need to be more widely available and accessible to provide comment to journalists [35].

Journalists are faced with many barriers to producing high quality health stories including a lack of time and space, problems understanding complex statistics and medical terminology and difficulty in accessing expert opinions [8]. As the internet changes the way people access news, traditional forms of the media are also changing. Newspapers, radio and television news are losing audiences at a steady rate and the international trend has been for media outlets to reduce staff. This results in increased pressure on both journalists and editors to produce stories quickly, a situation where quality can become easily compromised [36]. The changing format of reporting, where stories are simultaneously used for traditional media as well as the internet, means journalists are called upon to comply with new timelines, as news websites are updated when news breaks, rather than the traditional evening broadcast or printing deadline.

Against this backdrop it is important that health reporting provides the public with accurate and unbiased information on the value of new medical treatments. Prospects seem mixed. While online news sources have improved their coverage of health topics the increased coverage provided by commercial current affairs programs is of extraordinarily poor quality, at least in Australia. If this is representative of the situation in the rest of the world, large sections of the population are being poorly informed or misinformed about treatments that potentially affect them and their families. This presents a challenge for all of us including science and medical journals and the researchers themselves.

## Supporting Information

Figure S1 Mean scores across media outlets (with SE bars) over four years.
(0.42 MB TIF)

Figure S2 Regression analysis* of average scores over the period of the study: online media only. *Score = 0.006xelapsed time (days)+44.301973; r^2^ = 0.015073 (P = 0.009); 95% Confidence Interval for slope 0.001514 to 0.010465
(1.16 MB TIF)
