# Supplementary figures and images for: Media Reporting of Health Interventions: Signs of Improvement, but Major Problems Persist
Source: PLoS One. 2009 Mar 18;4(3):e4831. doi: 10.1371/journal.pone.0004831 (PMC2652829; doi:10.1371/journal.pone.0004831)

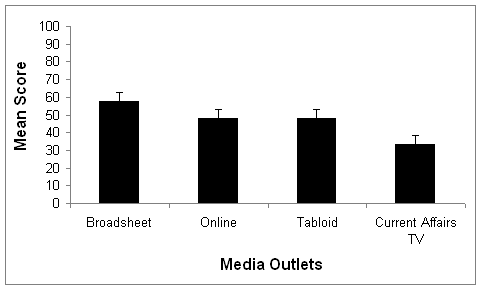

Supplement: Figure S1 — Mean scores across media outlets (with SE bars) over four years. (0.42 MB TIF) [file pone.0004831.s001.tif]

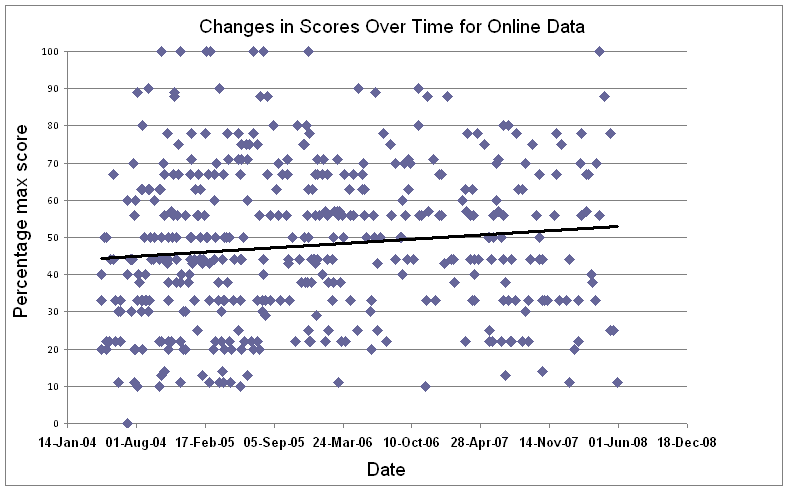

Supplement: Figure S2 — Regression analysis* of average scores over the period of the study: online media only. *Score = 0.006xelapsed time (days)+44.301973; r2 = 0.015073 (P = 0.009); 95% Confidence Interval for slope 0.001514 to 0.010465 (1.16 MB TIF) [file pone.0004831.s002.tif]
